# TGF-β1 and CD68 immunoexpression in capsules formed by textured implants with and without mesh coverage: a study on female rats

**DOI:** 10.1590/acb370201

**Published:** 2022-04-22

**Authors:** Ralf Berger, Jurandir Marcondes Ribas, Marcelo Augusto de Souza, Pedro Henrique de Paula, João Gabriel Cavazzani Doubek, Rafael de Castro e Souza Pires, Paulo Afonso Nunes Nassif, Eduardo Nascimento Silva

**Affiliations:** 1 Fellow PhD degree. Postgraduate Program in Principles of Surgery – Faculdade Evangélica Mackenzie – Curitiba (PR), Brazil.; 2 Associate Professor. Postgraduate Program in Principles of Surgery – Faculdade Evangélica Mackenzie – Curitiba (PR), Brazil.; 3 Medicine Graduate student – Universidade Estadual de Ponta Grossa – Ponta Grossa (PR), Brazil.; 4 Graduate student. Faculdade Evangélica Mackenzie – Curitiba (PR), Brazil.; 5 Plastic Surgeon. Regional University Hospital of Campos Gerais – Universidade Estadual de Ponta Grossa – Curitiba (PR), Brazil.; 6 Associate Professor. Faculdade Evangélica Mackenzie – Curitiba (PR), Brazil.; 7 PhD in Plastic Surgery. Universidade Estadual de Ponta Grossa – Ponta Grossa (PR), Brazil.

**Keywords:** Breast Implants, Mammaplasty, Surgical Mesh, Rats

## Abstract

**Purpose:**

To evaluate fibrosis formation and number of macrophages in capsules formed around textured implants without and with mesh coverage.

**Methods:**

Fibrosis was analyzed through transforming growth factor-beta 1 (TGF-β1) immunomarker expression and the number of macrophages through CD68 percentage of cells in magnified field. Sixty female Wistar rats were distributed into two groups of 30 rats (unmeshed and meshed). Each group was then subdivided into two subgroups for postoperative evaluation after 30 and 90 days. The p value was adjusted by Bonferroni lower than 0.012.

**Results:**

No difference was observed in fibrosis between meshed and unmeshed groups (30 days p = 0.436; 90 days p = 0.079) and from 30 to 90 days in the unmeshed group (p = 0.426). The meshed group showed higher fibrosis on the 90th day (p = 0.001). The number of macrophages was similar between groups without and with mesh coverage (30 days p = 0.218; 90 days p = 0.044), and similar between subgroups 30 and 90 days (unmeshed p = 0.085; meshed p = 0.059).

**Conclusions:**

In the meshed group, fibrosis formation was higher at 90 days and the mesh-covered implants produced capsules similar to microtextured ones when analyzing macrophages. Due to these characteristics, mesh coating did not seem to significantly affect the local fibrosis formation.

## Introduction

Autologous breast reconstruction techniques are cited as standard and present a higher general satisfaction rate in the long term^1^. However, autologous reconstruction in some patients might not be possible. For example, young women might not have enough abdominal tissue to enable the reconstruction using autologous techniques^2^.

In addition, some women might not be willing to accept the morbidity of the donor area, long surgery, long stay in hospital and recovery, which are inherent to the autologous reconstruction^3^. Therefore, implant reconstruction is an option in such cases. This procedure is associated with reduced complication rates and costs with hospital care^2^.

The major limitation of implant reconstruction is the unsuitable coverage of soft tissues, which might lead to skin damage, implant exposure, unsuitable esthetic results, and asymmetry^4^. One resource used to tackle the lack of tissue after oncological resection is the use of acellular dermal matrix (ADM), which provides an extra layer of coverage and support to the lower pole of the reconstructed breast^5^ and reduces the occurrence of capsular contracture^6^.

Although the ADM use is well established in the literature, the procedure presents high cost, which prevents its use in some circumstances. Therefore, the use of synthetic meshes, which cost is lower, might be an alternative^7^.

Better control of the implant pocket can be obtained by using a mesh^8^. However, the use of synthetic meshes in breast reconstruction creates new sceneries and requires that surgeons be able to recognize new complications and their histological behavior.

In relation to this issue, professionals are already aware that the periprosthetic tissues of breast implant capsules present higher transforming growth factor-beta 1 (TGF-β1) expression than healthy tissues^9^. The fibroblast stimulation by theTGF-β1 results in its conversion into α-SMA positive myofibroblasts and increases collagen synthesis and the contractile force^10^. This fibrotic process can be considered an important cause of capsular contracture^11^.

The cluster of differentiation 68 (CD68) is a protein expressed by cells in the monocyte lineage^12^. Therefore, it is related to chronic inflammation and giant cell reaction. In cases of severe capsular contracture, a substantial increase can be observed in the CD68 expression^13^.

Researching these markers in tissues of implant capsules and meshes enables the quantification and comparison of the presence of fibrosis and histiocytes and, therefore, the prediction of a relation between the presence of the mesh studied and the occurrence of chronic inflammation and capsular contracture, which are undesired effects in breast reconstruction.

This study aimed to evaluate capsules formed around microtextured silicone implants, with and without polyester mesh (Parietex) coverage regarding the presence of fibrosis and macrophages, which would favor the formation of capsular contracture or not.

## Methods

The study was carried out in the vivarium and in the Laboratory of Operative Technique and Experimental Surgery, Universidade Estadual de Ponta Grossa (protocol numbers 13.252/2018 and 3.973/2018), after being approved by the Ethics Committee on the Use of Animals (CEUA), process number 032/2018.

This is a prospective, non-randomized, interventional primary study. No calculations were performed for the sample size, obtaining a relatively small sample based on already published articles, similar to this one, favoring the process of acceptance by the CEUA.

Sixty albino rats (*Rattus norvegicus*) of the Wistar strain, weighing between 200 and 300 g, 100 days old were used. The 60 animals were distributed into two groups of 30 rats each (meshed and unmeshed implants), and each group was divided into two subgroups, to be evaluated at 30 and 90 days. Four rats were allocated per 450-cm^3^ acrylic box, lined with wood shavings. They had free access to water and specific diet for the species, ad libitum, in addition to alternating light in 12-h cycles at room temperature.

By the date of the first euthanasia, on day 30, eight animals in the unmeshed group and five in the meshed group died. One animal from each group was excluded due to the lack of quality of the piece and two animals from the meshed group by rotation of the mesh-implant set. After that, the following distribution was carried out (Table 1).

**Table 1:** Final distribution of animals in groups and subgroups.

Groups	Subgroups	
	30 days	90 days
Unmeshed	10 animals	11 animals
Meshed	10 animals	12 animals

### Implanted materials

LifeSil (Curitiba/PR, Brazil) implants were used, which have the same characteristics as microtextured implants, except that they are not filled with silicone, constituted only by the 20-mm-diameter microtextured implant cover.

The Parietex Composite (Medtronic, Minneapolis, USA) mesh, used to cover the outer surface of the implants in one of the groups, consisted of three-dimensional multifilament polyester with an absorbable, continuous and hydrophilic film on one side. The film consisted of porcine collagen, polyethylene glycol, and glycerol.

### Surgical procedure

The rats were anesthetized with intraperitoneal injection of ketamine 10%, 80 mg/kg, and xylazine 2%, 10 mg/kg. No fasting was performed, and they were placed in prone position after trichotomy.

A 1.5-cm-long incision was made in the posteroinferior costal margin, in the midline. The implant pocket was round and the implants were positioned 5 mm above the incision. On the meshed implants, the matrix was positioned on the dorsal side. The suture was performed with four stitches, Prolene 5.0 (Ethicon, New Jersey, United States), and there were no dressings.

Postoperative analgesia was performed with two subcutaneous doses of ketoprofen 5 mg/kg, with an administration interval of 24 h.

Euthanasia was performed with three times the therapeutic dose of ketamine (240–270 mg/kg) and xylazine (30–40 mg/kg)intraperitoneally, followed by cervical dislocation.

### Immunohistochemical method

Concentrated antibodies were diluted, following optimal dilution found in previous tests, applied and incubated in a moist chamber (Table 2).

**Table 2:** Antibodies and their dilution.

Primary antibody	Cell location	Clone	Brand	Standardized dilution
TGF-β1	Cytoplasmic membrane	E11262	Sprin	1:200
CD68	Cytoplasmic membrane	KP-1	BioSB	1:400

**Figure 1:**
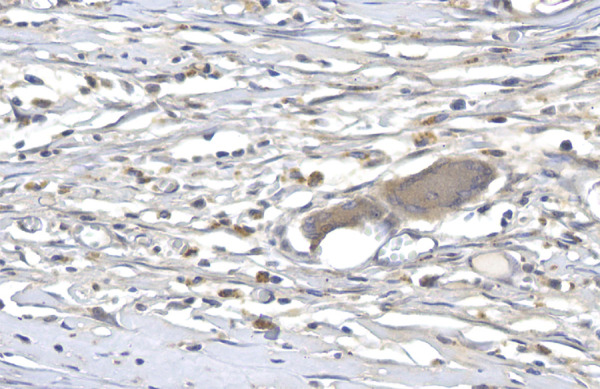
Photomicrography showing the TGF-β1-markedcapsular tissue (400× magnification, no polarized light).

Note: brown stained tissue TGF-β1 indicating the areas of fibrosis.

Immunomarked slides were digitalized using the slide scanner Axio Scan.Z1 (ZEISS, Jena, Germany). From the resulting digitalized file, between 10 and 15 images were generated using the software ZEN Blue 3.1 (ZEISS), representing the areas that adhered to the implant in the portion adjacent to the dermis (Fig. 1).

For quantification of the areas related to the immunohistochemical markers, the semiautomated color segmentation tool was employed, aided by the software Image-Pro Plus 4.5 (Media Cybernetics, Rockville, USA), with the purpose of determining the colors representing the immunoexpression and total tissue areas. The areas were artificially marked with red for immunoreactive regions and green for total tissue, aiming to standardize the value of the areas of structures of each image. Such color standardization was saved in a file named *mask*.

The mask was applied to all images, resulting in the area (square micrometers) of the two regions marked. The area values were exported to an Excel spreadsheet (Microsoft, Washington, USA).

### Immunohistochemical markers

**Figure 2:**
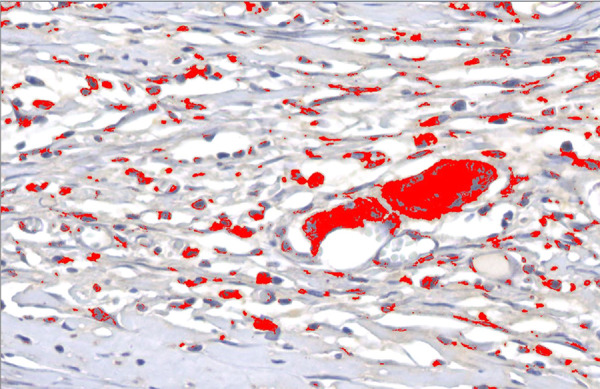
Photomicrography after mask application by the software image pro-plus (400× magnification, no polarized light).

Note: Positive expression of the immunomarker in red indicating fibrosis formation.

For each of the markers (TGF-β1 and CD68) within each subgroup (30 and 90 days), the groups (meshed and unmeshed) were compared regarding positivity average/total area. Next, within each group (meshed and unmeshed), the subgroups were compared (30 vs. 90 days) (Fig. 2).

### Statistical analysis

The results obtained in this study were described as means, standard deviation, medians, and minimum and maximum values (quantitative variables). To compare the groups (meshed and unmeshed) and subgroups (30 and 90 days), in relation to the TGF-β1 and CD68 markers expression, the nonparametric Mann–Whitney test was used. The normality condition of the continuous quantitative variables was evaluated employing the Shapiro–Wilk test. The significance level adopted was 0.05, submitted to Bonferroni correction in multiple comparisons (p < 0.012 values indicated statistical significance). Data was analyzed using the computer program Stata/SE v.14.1 (StataCorpLP, USA).

## Results

### Fibrosis

**Figure 3:**
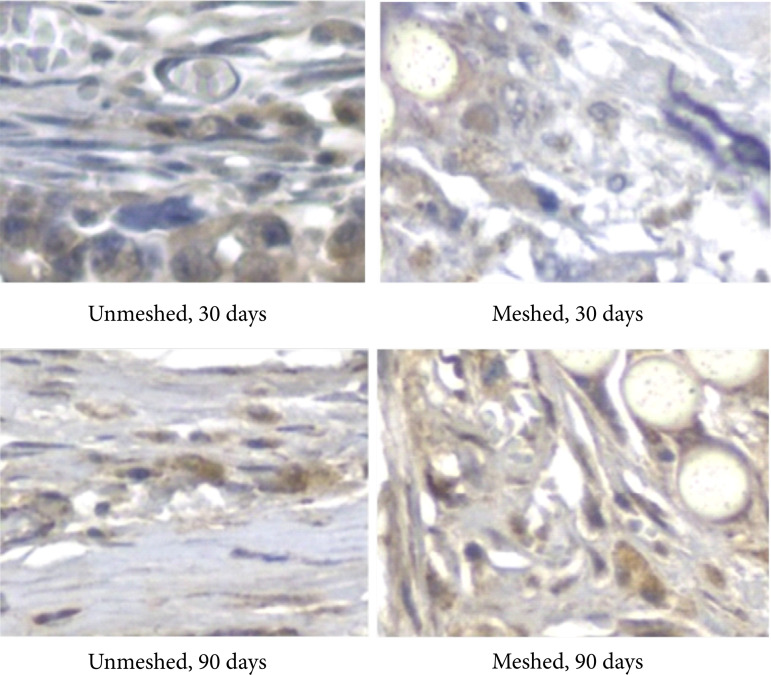
Photomicrography of the textured implant capsule with mesh coverage.

Note: brown stained TGF-β1 indicating fibrosis formation, in each group and subgroup, no polarized light (400×).

**Figure 4:**
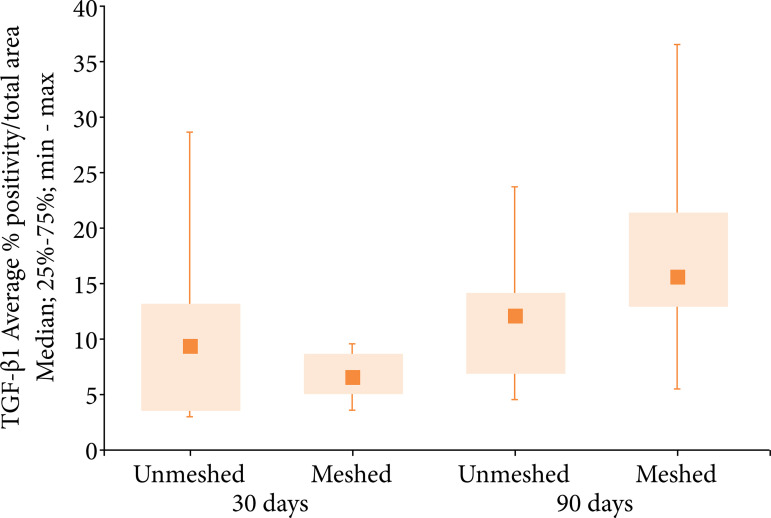
Medians, quartiles, minimum and maximum values of the TGF-β1 expression according to the group (meshed and unmeshed) and subgroups (30 and 90 days)

When fibrosis formation was analyzed through intensity of TGF-β1 (Fig. 3) in the meshed and unmeshed groups, no statistical difference was observed (Fig. 4, Tables 3 and 4). The meshed group showed more fibrosis formation at 90 days (Table 3 and Fig. 4).

**Table 3:** Comparison of the TGF-β1 positive area percentage average in both groups and in each subgroup.

Group	Subgroup	n	TGF-β1 AVERAGE % POSITIVITY/TOTAL AREA				
			Average	Median	Minimum	Maximum	Standard Deviation
Unmeshed	30 days	10	10.6	9.0	3.1	28.7	7.9
Meshed		10	6.8	6.5	3.6	9.7	2.1
Unmeshed	90 days	11	12.2	12.2	4.6	24.1	6.0
Meshed		12	17.7	15.9	5.5	36.7	8.8

When comparing the formation of fibrosis (TGF-β1 positive area percentage values), the group with mesh presented significant difference between subgroups (Table 4).

**Table 4:** Comparison of TGF-β1 positivity/total area percentage in each group and between subgroups.

Group/Subgroup	Comparison	p*
30 days	Unmeshed vs. meshed	0.436
90 days	Unmeshed vs. meshed	0.079
Unmeshed	30 days vs. 90 days	0.426
Meshed	30 days vs. 90 days	0.001

* Mann–Whitney nonparametric test, p < 0.012 (Bonferroni correction).

### Macrophages

**Figure 5:**
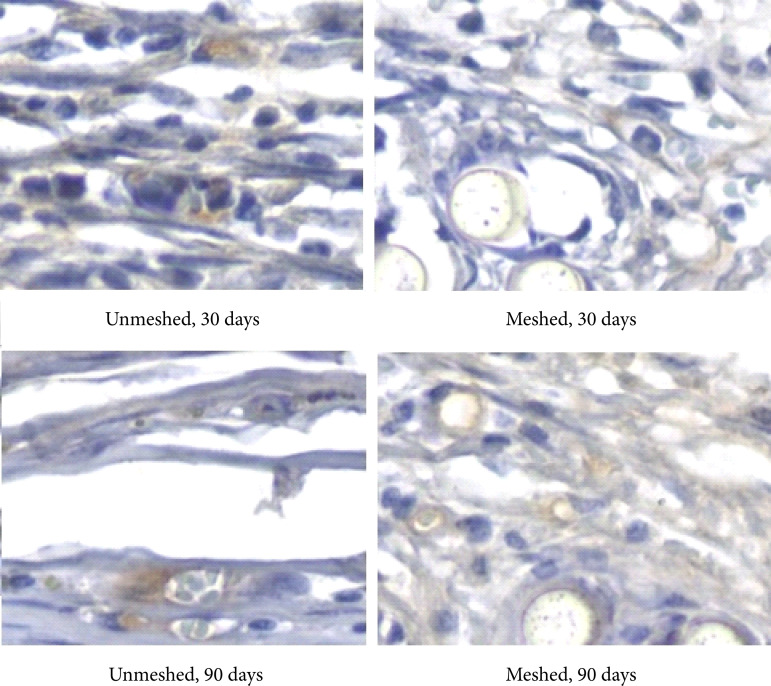
Photomicrography of the textured implant capsule with mesh coverage.

Note: brown stained CD68 indicating number of macrophages in each group and subgroup, no polarized light (400×).

**Figure 6:**
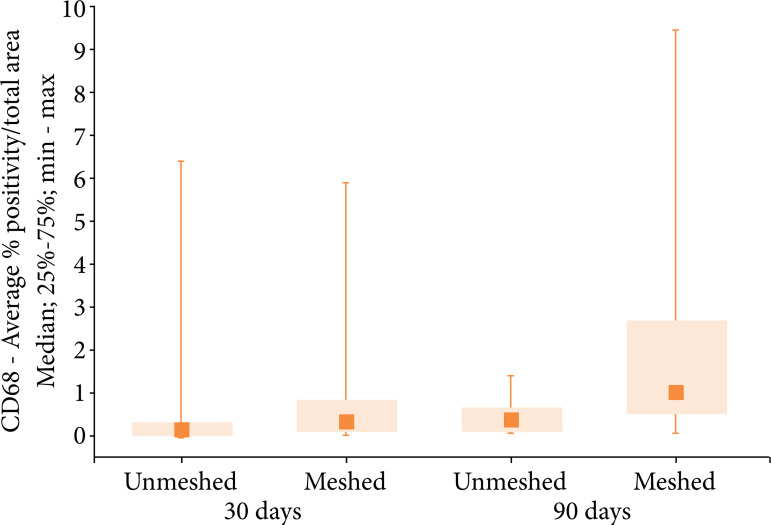
Medians, quartiles, minimum and maximum values of the CD68 expression according to the group (unmeshed and meshed) and subgroups (30 and 90 days).

In the statistical analysis of the number of macrophages, analyzed through the CD68 imunnomarker expression intensity (Fig. 5), statistical similarity was observed between the groups and subgroups analyzed (30 and 90 days) (Fig. 6, Tables 5 and 6). When comparing the number of macrophages (CD68 positive area percentage values) in each group and all subgroups, no statistical difference was found.

**Table 5:** Comparison of the CD68 positive area percentage in both groups and in each subgroup.

Group	Subgroup	n	CD68-AVERAGE % POSITIVITY/TOTAL AREA				
			Average	Median	Minimum	Maximum	Standard deviation
Unmeshed	30 days	10	1.02	0.05	0.01	6.47	2.12
Meshed		10	0.91	0.29	0.02	5.95	1.80
Unmeshed	90 days	11	0.52	0.39	0.10	1.45	0.47
Meshed		12	2.20	1.01	0.03	9.48	2.78

**Table 6:** Comparison of CD68 positivity/total area percentage values in each group and between subgroups.

**Group/subgroup**	**Comparison**	p*
30 days	Unmeshed vs. meshed	0.218
90 days	Unmeshed vs. meshed	0.044
Unmeshed	30 days vs. 90 days	0.085
Meshed	30 days vs. 90 days	0.059

* Mann–Whitney nonparametric test, p < 0.012 (Bonferroni correction).

## Discussion

Literature regarding the structural similarity between rats and human beings justifies the use of this species in studies on healing and implant capsules. Aiming at reproducibility of results, this study employed female rats (*Rattus norvegicus albinus*). The date of the animals’ euthanasia was chosen based on its correspondence to the human age. In rats, 90 days correspond to approximately 10 years of age for humans^14^.

Due to the difficulty to obtain large animal samples for research, sample size was based on already published articles similar to this one, which also used animal models. Thus, no calculations were performed for the sample size, obtaining a relatively small sample, facilitating the process of acceptance by the CEUA. Since it is a small sample, there may have been loss of statistical power in the analysis of the variables. Despite differing percentages in their values, significance range are stipulated (p < 0.012 with Bonferroni correction) to complete the analysis, even though there is an increased chance of false negative results.

Dermal matrix or meshes are mainly used in women that present lack of muscle coverage, since a full coverage of the implant/mesh set would probably be impossible^7^. To simulate the retroglandular pocket, subcutaneous dissection superficial to the *panniculus carnosus* was carried out on the back of the rats.

The use of meshes of different materials has been widely employed in immediate breast reconstruction with silicone implants^15^. Better control of the implant is achieved with the mesh, minimizing the problem of malpositioning due to the larger amount of tissue available to cover the implant^8^.

Healing occurs through several phases in which cytokines and biochemical markers are released characterizing each phase of the tissue repair^16^. The physiological response to the implant is started by polymorphonuclear leukocytes that secrete leukotriene and inflammatory mediators that stimulate fibroblast migration and proliferation. In addition, mastocytes, platelets and macrophages secrete TGF-β, which favor fibroblast differentiation into myofibroblasts resulting in collagen synthesis^10^^,^ 17–19.

Bui *et al.* 20 concluded that capsular contracture includes capsule thickening, collagen fiber alignment, and higher amount of myofibroblasts. This fibrotic and contractile character helps in the aforementioned complication.

Another important healing immunomarker is CD68, a protein expressed by cells of the monocyte lineage, including tissue circulating and resident macrophages^12^. In severe capsular contracture, the level of this immunomarker is typically increased^13^. It is due to the fact that, in addition to the fibrotic process, capsular contracture has a large part of an inflammatory character, here represented by macrophages.

Positive CD68 histiocytes produce cytokines and growth factors that stimulate fibrocytes, which migrate to tissue injured areas. Fibrocytes differentiate into fibroblasts that produce type III collagen and play an important role in fibrosis^21^.

Therefore, when seeking alternatives to ADM, macrophages amount (CD68 expression) similar to the implant coverage would be desirable, since capsular contracture is related to thicker capsules with higher amount macrophages^13^^,^ 20. Greater capsule thickening, higher collagen fiber alignment and the presence of contractile myofibroblasts are characteristics of capsular contracture^20^.

The results of this study disagree with findings reported by Vieira *et al.* 22, who compared textured implants to polyurethane implants and found higher TGF-β expression in implants with polyurethane coverage. It seems relevant to mention that those authors used polyurethane as an additional layer to the implant texture, while this study employed Parietex mesh.

Steiert *et al.* 23 studied silicone implants in rats by activating their surface covalently with anti-FAS immunosuppressor antibodies with the purpose of suppressing the foreign body reaction. They found lower TGF-β and CD68 expression when using the anti-FAS, demonstrating that this fibrosis marker reduces when the immune response is lower, which points out the importance of the implant surface in the fibrosis outcome.

This study is in agreement with Ludolph *et al.* 24, who found, at 84 days, similar TGF-β1 expression between the textured implant group and the implant and porcine ADM group. Those researchers compared textured implants to implants covered with porcine dermal matrix.

Results obtained by Lombardo *et al.* 25 showed thinner capsules with lower TGF-β2 expression in a study on capsular contracture in rats in which omega-3 was used. This substance has been reported as an inhibitor of the production of a wide range of pro-inflammatory eicosanoids. This result reinforces the link between inflammation, capsule thickness and the TGF-β family expression.

Unlike Pontes *et al.* 26, who compared nanotextured implants to polyurethane implants and found lower TGF-β and CD68 expression in the nanotextured implants, this study found similar TGF-β expression between unmeshed and meshed groups. Those authors also found higher TGF-β expression at 60 days than at 30 days in the nanotextured group, while this study observed stability in this marker expression in the textured group, increasing the expression from the 30^th^ to the 90^th^ day only in the meshed group.

Also, in the polyurethane group, Pontes *et al.* 26 found higher CD68 expression at 60 days than at 30 days, while in this study, CD68 was not altered. It might be relevant to mention that those researchers compared nanotextured implants to implants with an additional layer (polyurethane), while this study compared microtextured implants to meshed implants.

The results of the present study partially disagree with those by Fisher *et al.* 27, who compared smooth to textured implants in rats and found lower marker expression in smooth implants at 60 days. This difference was not observed at 120 days. However, they compared different silicone coverage not meshes as in our study.

The results of this study confirm those found by Bui *et al.* 20, who studied capsules removed from patients with different postoperative times and found no differences in the CD68 expression in relation to the postoperative time.

Finally, this study does not agree with the report put forward by Ludolph *et al.* 24, who compared unmeshed and meshed silicone implants using acellular porcine dermis and found lower CD68 expression in the dermal matrix group at 21 days. At 84 days, they observed an inverse pattern, with higher CD68 expression when the implant was covered by the matrix. In this study, at 30 and 90 days, the CD68 expression was similar between the groups.

However, further studies are still needed to evaluate the histological behavior of the several meshes available in the marked associated to silicone implants.

## Conclusions

In the meshed group, the fibrosis formation was higher at 90 days.

The mesh-covered implants produced capsules similar to the microtextured ones when analyzing the number of macrophages.

Due to these characteristics, mesh coverage did not seem to significantly affect the local chronic inflammation and fibrosis formation.
